# Applications of Lightsheet Fluorescence Microscopy by High Numerical Aperture Detection Lens

**DOI:** 10.1021/acs.jpcb.4c01721

**Published:** 2024-08-23

**Authors:** Chun-Pei Shih, Wei-Chun Tang, Peilin Chen, Bi-Chang Chen

**Affiliations:** † Institute of Physics, Academia Sinica, Taipei 11529, Taiwan; ‡ Department of Chemistry, National Taiwan University, Taipei 106319, Taiwan; § Nano Science and Technology Program, Taiwan International Graduate Program, Academia Sinica and National Taiwan University, Taipei 11529, Taiwan; ∥ Research Center for Applied Sciences, Academia Sinica, Taipei 11529, Taiwan

## Abstract

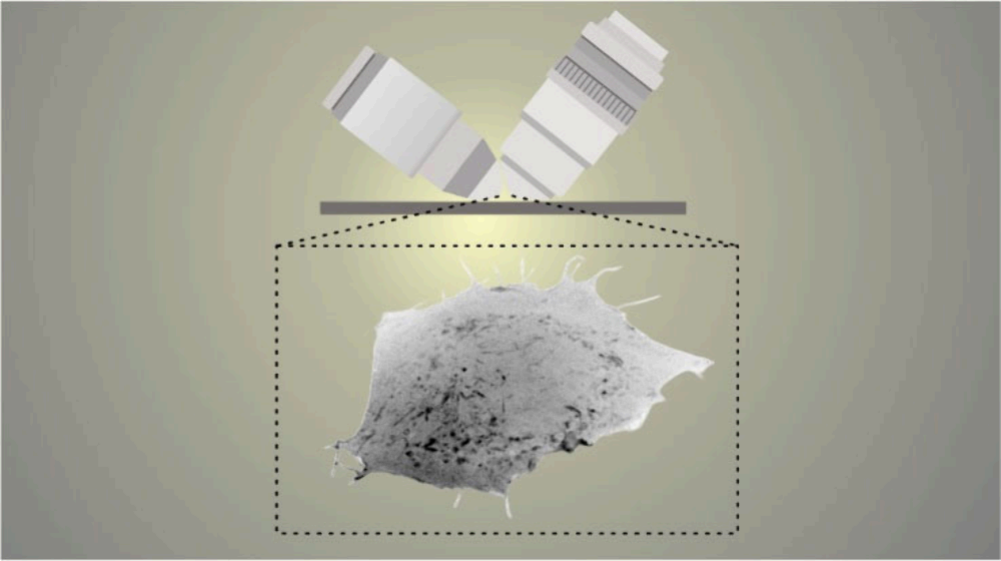

This Review explores the evolution, improvements, and recent applications of Light Sheet Fluorescence Microscopy (LSFM) in biological research using a high numerical aperture detection objective (lens) for imaging subcellular structures. The Review begins with an overview of the development of LSFM, tracing its evolution from its inception to its current state and emphasizing key milestones and technological advancements over the years. Subsequently, we will discuss various improvements of LSFM techniques, covering advancements in hardware such as illumination strategies, optical designs, and sample preparation methods that have enhanced imaging capabilities and resolution. The advancements in data acquisition and processing are also included, which provides a brief overview of the recent development of artificial intelligence. Fluorescence probes that were commonly used in LSFM will be highlighted, together with some insights regarding the selection of potential probe candidates for future LSFM development. Furthermore, we also discuss recent advances in the application of LSFM with a focus on high numerical aperture detection objectives for various biological studies. For sample preparation techniques, there are discussions regarding fluorescence probe selection, tissue clearing protocols, and some insights into expansion microscopy. Integrated setups such as adaptive optics, single objective modification, and microfluidics will also be some of the key discussion points in this Review. We hope that this comprehensive Review will provide a holistic perspective on the historical development, technical enhancements, and cutting-edge applications of LSFM, showcasing its pivotal role and future potential in advancing biological research.

## 1 Introduction

### 1.1 Light Sheet Fluorescence Microscopy

**Figure 1:**
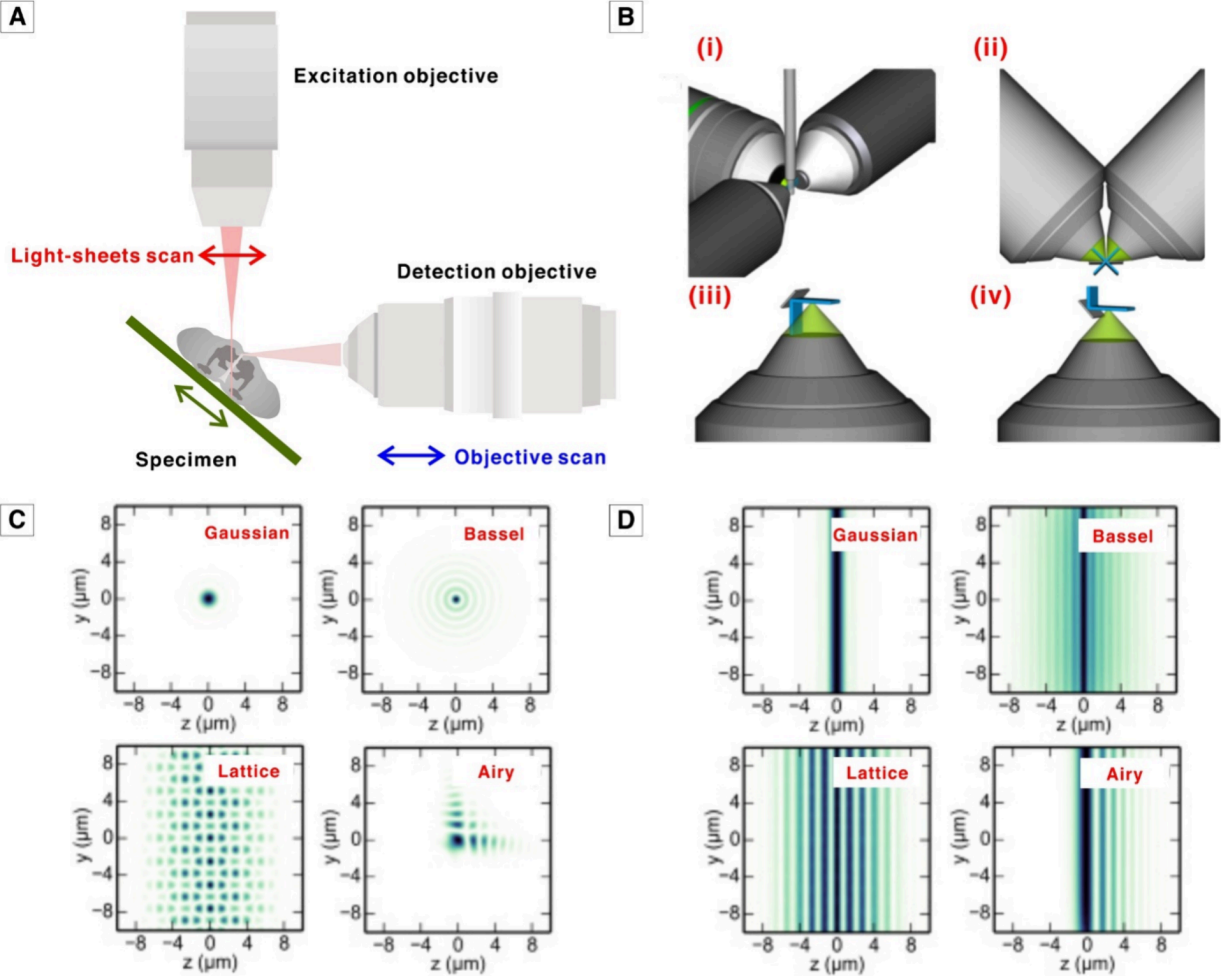
General light sheet fluorescence microscopy setup. (A) Typical setup schematic of light sheet fluorescence microscopy. **(B(i)–(iv))** Various optical geometry used in light sheet fluorescence microscopy. Light sheet is represented in blue and the detection cone in green. **(i)** Multiple objectives. **(ii)** Double side illumination and double side detection. **(iii)** Single objective lens and a micromachined mirror. **(iv)** Two en face objectives and an AFM tip mirror. (C) Excitation profile of various light sheet generation methodology. (D) Intensity profile of various light sheet generation methodology. **(B–D)** Adapted with permission from ref (^21^). Copyright 2018 Optica Publishing Group.

Light sheet fluorescence microscopy (LSFM) is a technique used to illuminate biological samples labeled with fluorophores, section by section, using a thin sheet of light.^1^ Typically, the light source generating the light sheet is positioned orthogonal to the detector (Figure 1A). As the specimen moves through the light sheet, signals are generated section by section optically until the entire three-dimensional (3D) structure is obtained.^2,3^ The freedom to manipulate the position of the objectives (Figure 1B) also enables analysis of specimens with varying sizes (animal embryos,^4^ whole mouse brains,^5^ tissues^6^). After the scanning is complete, the emitted signals are collected and processed by a computer to visualize the entire specimen in 3D with high spatial and temporal resolution. Due to the confinement of light sheet illumination, only a thin slice of the specimen (typically micrometer thickness required for subcellular imaging) is exposed, drastically reducing the chance of phototoxicity and photobleaching.^7^ This was demonstrated by Planchon et al. when they compared the effect of photobleaching on the image stacks using DSLM and confocal microscopy.^8^ Although LSFM may seem like a highly advantageous technique for modern-day biological imaging analysis, there are several challenges to be addressed throughout its development as a standalone device. In recent years, in order to overcome these challenges, the integration of LSFM with various techniques that tackle specific limitations such as restricted penetration depth or size constraints has provided possibilities for further improvements.^9^

### 1.2 Evolution of Light Sheet Fluorescence Microscopy

The discovery of ultramicroscopy by Siedentopf and Zsigmondy in the early 19th century laid down the fundamental principle of LSFM.^10^ In 1993, Voie et al. proposed a setup based on ultramicroscopy known as orthogonal plane fluorescence optical sectioning to visualize the internal structures of cochlea in rodents.^11^ A milestone for LSFM was reached in the year 2004 upon the success of whole living organisms imaging using a single-plane illumination microscope (SPIM).^12^ Since then, LSFM has become increasingly popular among researchers and many similar configurations have been developed for various applications. Digital scanned laser light sheet fluorescence microscopy, proposed by Keller et al. in the year 2008, was capable of producing high-quality images of large specimens with high-speed acquisition capabilities.^3,4^ Variations of existing designs that resolve issues regarding light scattering and absorption start to emerge a few years later. For example, the development of multiview light sheet microscopy (MuVi-SPIM) in 2012 by Krzic et al. was able to decrease image distortion by simultaneous recording of multiple 3D images from four different directions. After reconstructing the collected images, high-resolution images can be generated in real-time.^13^ Around the same time period (2010s), the performance of LSFM was improved by changing the methodology regarding light sheet generation, such as employing Bessel beam^8,14^ or lattice beam.^15^ In recent years, the development of LSFM has made remarkable progress with the integration of adaptive optics (AO)^16,17^ and multimodal imaging.^18^ There are also significant advancements in the area of data processing and analysis as the use of artificial intelligence (AI) becomes more common.^19^

### 1.3 Light Sheet Fluorescence Microscopy for Biological Research

Since the early 2000s, LSFM has become increasingly popular as an imaging tool for many biological researchers. Initially, low numerical aperture (NA) detection objectives were favored for their wide field of view and reduced photodamage in the imaging of large samples. However, the shift toward high NA detection objectives has revolutionized LSFM with enhanced resolution, which opens the door to subcellular imaging. For the scope of this review, we define detection objectives with NA higher than or equal to 1.0 as high NA. Any NA value lower than 1.0 is considered low NA. We also focus on a high NA detection objective (lens) to improve the optical sectioning capability at the expense of the field of view.

The utilization of high NA detection lenses provides precise visualization of cellular structures and dynamics within biological specimens^20^ as well as improved spatial resolution. To understand the important role of high NA detection objectives in biological research, we consider the following two formulas for resolution (transversal and axial).

Transversal resolution, *R*_*T*_, given by Rayleigh criteria as
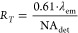
and axial resolution, *R*_*A*_, given by Rayleigh criteria as
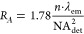
where NA_det_ is the NA of the detection objective, *n* is the refractive index, and λ_em_ is the emitted wavelength.^21^

From the formula, it was deduced that the NA of the detection objective greatly affects the axial resolution compared to the transversal resolution. For example, assuming the emitted wavelength is the same for both cases, the change of NA value from 0.8 to 1.1 in transversal resolution only slightly improves the resolution from 0.76 to 0.55. For axial resolution, the change in NA value from 0.8 to 1.1 improves the resolution from 2.78 to 1.47. This enhancement in axial resolution empowered researchers to explore biological samples from tissue-level morphology to intricate subcellular or submicron interactions, fostering a deeper understanding of biological phenomena.^22^ Even though high NA detection objective setups provide enhanced axial resolution, some notable disadvantages must be carefully evaluated. For instance, the physical constraints of using high NA detection objectives must be carefully evaluated. Most high NA detection objectives are bulky, which incurs difficulties during setup as well as during optical alignments for systems with high NA excitation objectives. Analysis of large samples will also be severely limited by the use of high NA objectives. Despite these disadvantages, high NA detection objectives are still favored in biological research, because the intricate details of subcellular entities cannot be revealed with low NA detection objectives. Recent developments also resolved the physical constraint issues by using a high NA single objective.^23,24^

As LSFM technology continues to evolve, high NA objectives have become more common in typical LSFM setups in biological research. Hardware and software barriers were overcome to resolve the problems brought forth by high NA detection objectives. Therefore, in this Review, we narrow our discussions to LSFM with high NA detection objectives and their biological applications.

## 2 Developments of Light Sheet Fluorescence Microscopy

In this section, we discuss various improvements of conventional LSFM setup in terms of hardware, software, and fluorescence probes used to enhance resolution, minimize phototoxicity and photobleaching, and increase image acquisition speed and data processing as well as sample handling. Under each type of improvements (hardware, software, fluorescence probe), specific upgrades/ideas are explained in detail, and relevant applications are provided to facilitate a comprehensive discussion.

### 2.1 Improvements in Hardware for High NA Applications

Since the upgrades of hardware enhance the overall performance of LSFM throughout its development, we first focus on the hardware of LSFM. Hardware such as light sheet generation systems, objective lenses, sample handling, and mounting devices were elaborated with respect to how they improve the overall performance of LSFM. The specific applications for different upgrades are also reviewed. We hope to provide readers with an overview of hardware improvements in recent years so that we can inspire the LSFM community to come up with more innovative designs.

#### 2.1.1 Light Sheet Generation

Light sheet generation is an integral and fundamental component of LSFM. Conventionally, a Gaussian beam is used to generate a light sheet in a standard LSFM setup.^25^ However, due to the limited depth of field, thick light sheets are produced, which may reduce the ability to selectively illuminate a single focal plane, hence leading to reduced optical sectioning, especially for subcellular imaging of cellular dimension.^26^ The introduction of Bessel beam plane illumination has provided a solution to deal with the challenge of generating thinner light sheets.^8,14^ Bessel beam plane illumination is characterized by the generation of the nondiffracting beam by projecting a ring-shaped light pattern onto the excitation objective lens, resulting in a beam with a narrow core and concentric side lobes.^27^ Owing to the self-reconstructing and nondiffracting properties, Bessel beams are heavily employed in LSFM for increasing depth of focus as well as reducing shadowing and scattering artifacts.^28^

Similar to the Bessel beam, another type of self-reconstructing and nondiffracting beam called the airy beam was also applied in light sheet generation.^29,30^ The airy beam possesses a unique intensity distribution profile that can be accounted for simple deconvolution, enabling efficient use of the acquired fluorescence signals without losing valuable information.^31^ Another popular technique to produce thinner light sheets is the use of an optical lattice. The technique involves the use of multiple laser beams positioned at different angles and a spatial light modulator (SLM) to produce an optical lattice, which was used to produce a thin and uniform light sheet along the propagation direction that greatly improves axial resolution and beam uniformity.^15^Figure 1C,D shows the excitation profile and intensity profile of various light sheet generation methodologies.

Being a fundamental component of LSFM, each type of light sheet offers varying properties that affect sample exposure, imaging depth, resolution, and phototoxicity. For example, Bessel beam and lattice beam possess the ability to minimize out-of-focus light, which enables improvement in contrast and decreases phototoxic effects.^32,33^ Another recent technique known as tiling light sheet was shown to enhance spatial resolution and imaging efficiency as compared to conventional light sheet.^34,35^ It is important to consider the specific imaging requirements for the desired applications that one has in mind, such as field of view or sample thickness, before choosing the most appropriate light sheet generation technique.

#### 2.1.2 Detection System

Over the years, the detection system in LSFM has undergone notable improvements, particularly in the transition from low NA to high NA detection objectives. During the early development of LSFM, low NA detection lenses were favored for wide-field imaging of larger samples such as zebrafish embryos^4^ or *Drosophila* embryos,^11^ prioritizing broad observation over high resolution. However, the need to probe deeper into subcellular structures pushes LSFM toward high NA detection objectives. This evolution has revolutionized LSFM’s imaging capabilities, facilitating enhanced resolution and improved imaging of subcellular structures. For instance, the shift to high NA detection lenses enables finer imaging details, allowing precise visualization of cellular dynamics within biological specimens.^36^ This progression signifies a pivotal advancement in LSFM, allowing researchers to explore biological samples with exceptional clarity, thus unveiling previously unseen intricacies within cellular structures.^37^

To overcome several physical constraints imposed on LSFM system when using high NA lenses, Theer et al. developed a system coined πSPIM that fully benefits from using objective lenses with high NA without trading off parameters.^38^ In addition, to accommodate the use of high NA when sampling large samples, Cai et al. designed a single-lens LSFM based on a Micro-Mirror Array that displays good axial resolution in large samples.^39^ Looking forward, we expect further development of high NA optics and imaging technologies that possess the potential to push the boundaries of resolution in LSFM.^40−42^ Integration to other platforms, such as AO,^43^ could also enhance the overall resolution of LSFM, thus further amplifying the ability to explore the complexities of biological systems.

#### 2.1.3 Sample Handling and Mounting System

One of the key reasons for the increasing popularity of LSFM is the fact that it is able to handle various biological samples. From *in vivo* imaging of live cells^44^ to defining the structure of a whole mouse brain.^5^ Therefore, the importance of sample handling and mounting systems must be realized. In a typical LSFM setup, the sample is moved simultaneously through the stationary light sheet. This can be done by placing the sample on an automatic stage that can be electrically controlled with high precision. There is also a scanning mode where the sample remains stationary while the light sheet and detection objective lenses move to capture the images.^21^ In this type of setup, the sample is kept stationary on a stable mounting system, while the position of the objective lenses is controllable. As the need to analyze more complex biological samples or fast dynamic processes increases in demand, scanning modes that involves simultaneous image acquisitions from different views^45^ and different focal planes^46^ were also becoming increasingly popular.

Other than the manipulation of lenses and automatic stages, it is also important to maintain a suitable environment and restrict the movements for live samples. Generally, it is common practice to use low melt agar to mount large specimens.^47^ However, this method does not work for all organisms. As pointed out by Burnett et al., small organisms such as *Caenorhabditis elegans* can burrow through the soft agar, lowering time-lapse imaging duration significantly.^48^ Another point of concern is that the gelling temperature for the low melt agarose might not be suitable for all organisms of interest.^49^ With the prior introduction of fluorocarbon foil (fluorinated ethylene propylene, FEP) for LSFM,^50^ Smith et al. proposed a mounting and immobilization protocol for the imaging of postembryonic *C. elegans* by using a refractive index matched, ultraviolet-activated adhesive hydrogel BIO-133 and FEP tube encasement. They successfully prolonged the imaging time of *C. elegans* and demonstrated the application of such protocol in other biological entities.^51^ There are also innovative custom designs of the mounting platform or sample chambers that were built to satisfy the needs of specific applications.^52−54^ One such design is the open-top dual-view and dual-illumination light sheet microscope presented by Moos et al. that was shown to analyze living large samples at single-cell resolution with high-throughput capability.^55^ We have seen the importance of designing suitable sample handling and mounting systems in LSFM to match specific applications. Even though the use of a universal system is often sufficient, one should pay close attention to the requirements in order to minimize the chance of artifacts and other potential issues since the nature of every biological specimen is different.

### 2.2 Improvements in Data Acquisition & Processing

The images obtained by LSFM are section by section. To construct 3D images, it relies on image processing and reconstruction, big data analysis, and various AI applications. We will discuss the transformation of data processing from two-dimensional (2D) to 3D scale and list some common programs that are used for processing. We also highlighted several applications that involve the integration of AI into LSFM systems, since AI becomes more and more important in the scientific community.

#### 2.2.1 Image Processing and Reconstruction

**Figure 2:**
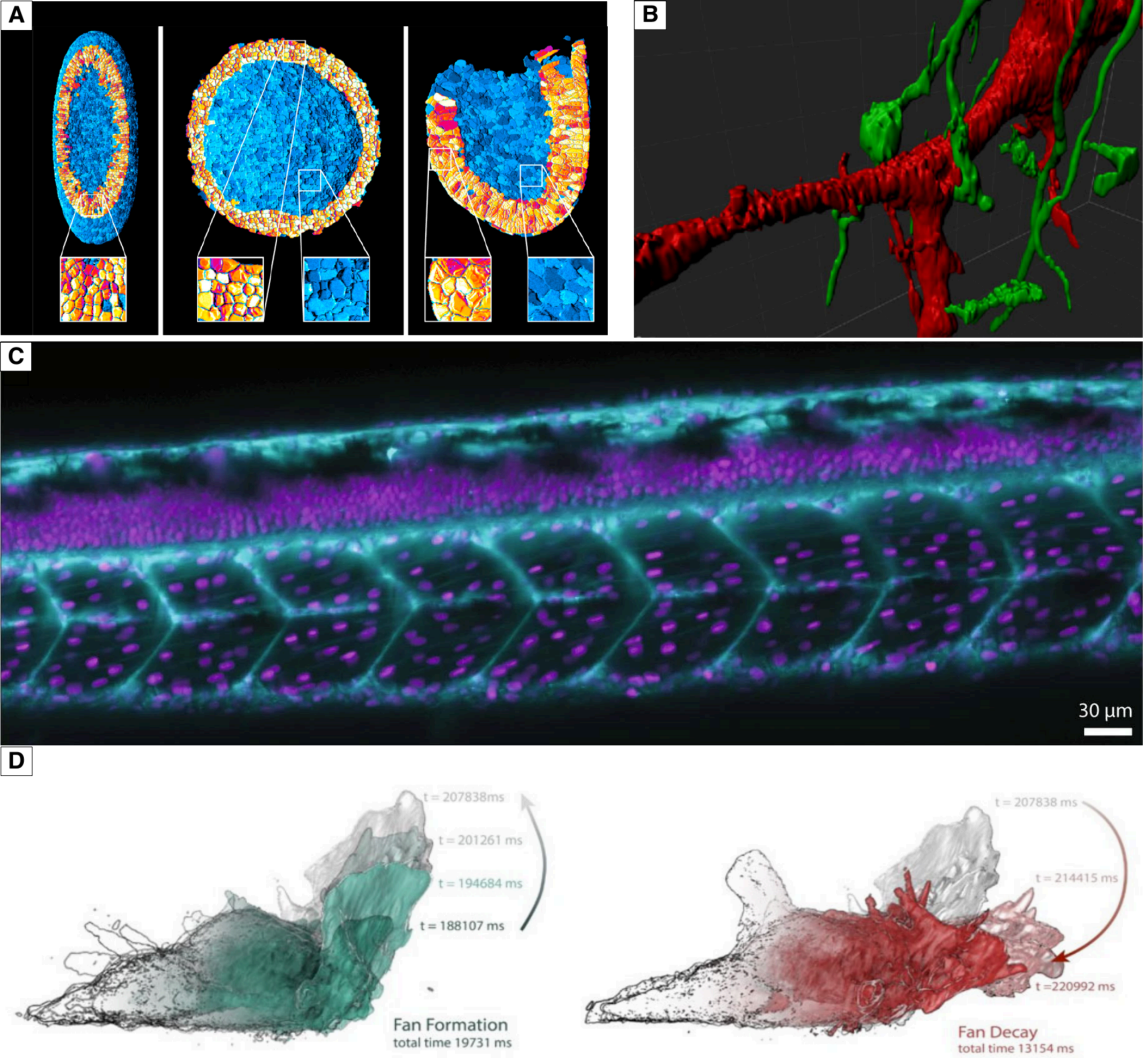
Image processing programs used to generate various sample images. (A) Image processing program: Packaged C++ applications. Sample: Whole-embryo cell of fruit fly, zebrafish, and mouse. (From left to right) Images show the whole-embryo cell segmentation results visualized as renderings of sliced embryos. Cells in the exposed cross sections are shown in an orange/red color scheme and in a cyan/blue color scheme for the rest of the embryo. Insets show enlarged views of the cell segmentation results. Reproduced with permission from ref (^59^). Copyright 2016 Elsevier Inc. (B) Image processing program: Custom written MATLAB scripts. Sample: Mossy fiber and parvalbumin cell. Image shown is the two color imaging of segmented parvalbumin cells and mossy fibers reconstructed in 3D. Reproduced with permission from ref (^60^). Copyright 2019 SPIE. (C) Image processing program: Napari. Sample: Zebrafish larvae. Napari was first used for multidimensional visualization and 3D rendering. High-resolution dual color magnified image of zebrafish larvae were produced after further processing. The larvae are imaged at 2 dpf. The nuclei (magenta) are labeled with tg(h2afva:h2afva-mCherry). The membranes (cyan) are stained using Vybrant DiO cell-labeling solution (Thermal fisher V22889). DiO injections for retrograde live label were applied at 24 hpf, followed by an O/N incubation at 29 °C incubator before imaging. Reproduced with permission from ref (^36^). Copyright 2022 Springer Nature. (D) Image processing program: UCSF Chimera. Sample: Neutrophil. Fast-moving neutrophils were characterized and analyzed with LSFM via UCSF Chimera program. The three-dimensional visualization enabled automated pseudopod detection which provided crucial information regarding pseudopod formation and cell turning.

Image processing and reconstruction play a vital role in LSFM, serving as a bridge that connects raw optical data to comprehensive 3D representations of biological samples. As the need to visualize increasingly complex samples, the data grow exponentially. The transition from 2D to 3D analysis involves capturing a stack of images along the *z*-axis to reconstruct a volumetric data set that is directly proportional to the number of optical sections acquired to build the 3D representation. The need for high-resolution images further contributes to the volume of the data. In order to process the vast amount of data generated by LSFM, there are many potential solutions offered by commercial companies and open-source software. Some examples are java-based ImageJ/FIJI community,^56−58^ packaged C++ applications,^59^ MATLAB,^60^ and python libraries.^61,62^Figure 2A,B shows some examples of the images generated with image processing programs, such as packaged C++ applications and custom-written MATLAB scripts. One particularly useful Python library, Napari (Figure 2C), can be used to view and explore 2D and beyond arrays on a canvas. Image visualization, annotation, and analysis can all be conducted via this library.^63^ There were even hybrids of different programming languages and libraries (UCSF Chimera, Figure 2D) that were developed for enhanced visualization.^64^ Custom-made software (VesselExpress) for specific analysis such as 3D data of blood vessel system can also be found online.^65^ When designing a robust image analysis pipeline involving LSFM, one should consider the guidelines provided by Gibbs et al., to avoid unnecessary data wrangling/resaving.^66^

Another important aspect of image processing in LSFM is deconvolution. The images generated by LSFM are often prone to issues like scattering and blurring, which lower the overall image quality. Deconvolution algorithms were applied to improve the contrast and resolution of images, thus restoring sharper details and enhancing overall image fidelity.^67,68^ As multiview imaging in LSFM becomes a common practice, deconvolution became a necessary tool to enhance resolution.^55,69,70^ Throughout the development of various deconvolution algorithms, many questions were raised on the approximations regarding point spread function (PSF) characteristics, which limits their use in advanced techniques such as particle tracking.^71^ However, recent studies provided solutions to overcome such issues by proposing new reconstruction methods,^72,73^ completely removing the need for deconvolution,^74^ or introducing computation algorithms such as compressed-sensing computation to recover a high-quality signal from a single incomplete measurement.^75^ There is even a deconvolution method that involves the use of deep learning,^30^ which will be discussed in detail in a later section.

#### 2.2.2 Big Data Analysis

The growing need for big data analysis in LSFM necessitates efficient data compression and storage strategies due to the substantial volume of information generated. To manage this influx of data, compression techniques tailored for volumetric data sets are crucial. These techniques’ goal is to decrease data size while preserving essential information and employing algorithms optimized to remove spatial and temporal redundancies present in LSFM acquisitions. Walker et al. compared several compression algorithms using a collection of published and unpublished data sets and determined the best software to be used for various microscopy applications, including LSFM.^76^ Balazs et al. proposed a real-time compression library that enables high-speed compression and decompression of data sets during acquisition. This algorithm also included a lossy option that yields a compression ratio of up to 100-fold.^77^ As mentioned earlier, it is also important to come up with effective storage strategies to accommodate the large quantities of image stacks that were produced during the experiments. Moore et al. proposed a common metadata format that can be applied in most bioimaging applications.^78^ Hence, by implementing streamlined data compression and storage practices, researchers can navigate the challenges posed by LSFM’s substantial data output, facilitating easier accessibility, analysis, and retrieval of invaluable biological insights.

#### 2.2.3 Artificial Intelligence

The recent leap in the development of AI has sparked excitement in the scientific community all over the world. The extensive use of machine learning and deep learning techniques has benefited many research areas. LSFM has begun to embrace AI to revolutionize image analysis and interpretation. The integration of machine learning algorithms is beneficial to many LSFM researches, such as the automation in the analysis of the entire mouse brain vasculature,^79^ imaging rare and complex cellular events like kinetochore dynamics during mitosis,^80^ and multidimensional analysis of lattice light sheet microscopy data.^81^ Deep learning, a subset of machine learning, has shown potential in LSFM by enabling the extraction of intricate patterns and features from complex 3D data, allowing for more accurate identification and characterization of biological structures. For example, Pan et al. were able to identify micrometastases and single cancer cells in full-body 3D scans with DeepMACT, which were also used to indicate the tumor microenvironment affects drug targeting efficacy.^82^ Schoppe et al. use an integrated pipeline to segment major organs and the skeleton in volumetric scans of mice without any human intervention or parameter tuning.^83^ Last but not least, Nehme et al. successfully demonstrated their approach, DeepSTORM3D, a designed optimal PSF for multiemitter, can be used to study biological processes in whole cells.^84^ As the technology in AI continues to evolve, we could foresee that more AI-driven tools can unlock new frontiers in our understanding of biological structures and dynamics.

### 2.3 Improvements in Fluorescence Probe

**Figure 3:**
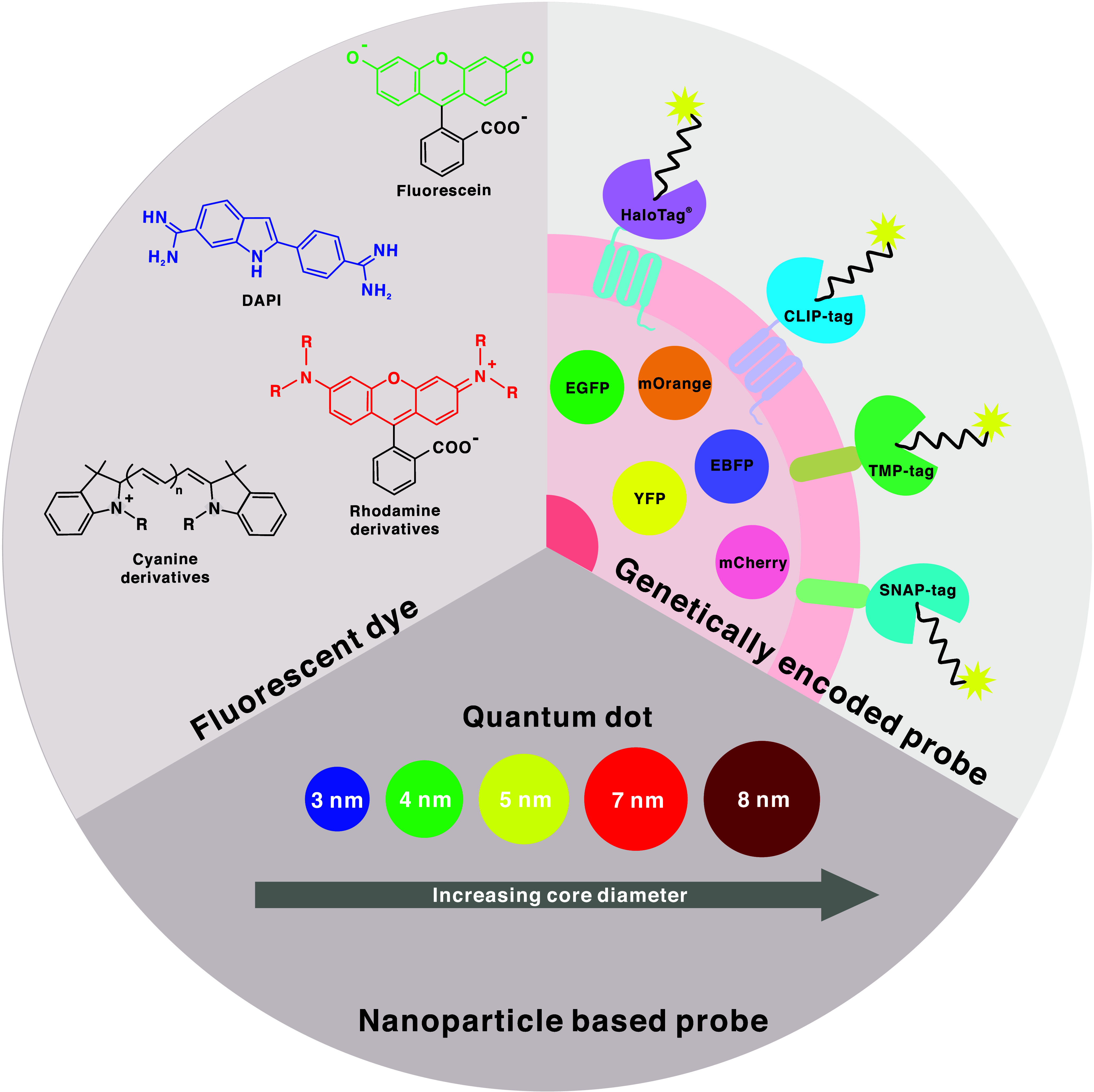
An overview of fluorescence probes commonly used in LSFM. Fluorescent dyes such as Fluorescein, Rhodamine derivative, Cyanine derivative, and DAPI. Genetically encoded probe such as EFGP, EBFP, YFP, mCherry, and mOrange. Protein tags such as HaloTag ^©^, CLIP-tag, TMP-tag, and SNAP-tag. Nanoparticle-based probes include quantum dots of varying sizes that display different spectral characteristics.

Another issue that determines the image quality is the amount of collected light from biological samples, which often relies on fluorescence probes. We discuss the development of fluorescence probes that greatly improve their optical properties in terms of brightness, quantum yield, photostability, and spectral diversity. Traditional fluorescence dyes and other types of fluorescence probes, such as those with specific targeting capabilities or genetically encoded probes, will also be mentioned. The Nobel prize-winning quantum dot research is also included under the discussion of nanoparticle-based probes. Figure 3 gives an overview of fluorescence probes commonly used for LSFM-related applications. There will also be a section that discusses integrated strategies that might be beneficial to LSFM-related applications in the future.

#### 2.3.1 Fluorescent Dyes

Fluorescent probes have always been an essential part of LSFM from its early developmental stage even until recent advancements. During early development, traditional fluorescent dyes such as fluorescein,^85^ rhodamine derivatives,^86^ or DAPI^87^ were used frequently. These small organic molecules can undergo bioconjugation with biomolecules of interest without interfering with its biological functions.^88^ Upon excitation, these dyes emit fluorescence, which enables visualization of the labeled biomolecules. More universal fluorescent dyes became commercially available in recent years, such as cyanine dyes^89^ or Alexa Fluor dyes.^90^ However, as LSFM continues to strive, there is an increased demand for the need to probe deeper into thick tissues and analyze complex specimens as well as large samples. Therefore, there is a need to push for the development of fluorescent probes to keep up with the pace of LSFM development. For instance, one of the important properties for fluorescent probes to improve upon is brightness. It is advantageous in many aspects to have a brighter fluorescent probe, because it directly impacts the quality, sensitivity, and depth of imaging. For example, Hammers et al. developed a bright fluorescent probe that has high specificity for H_2_S, which enabled a rapid fluorescence signal enhancement when stimulated by H_2_S and was used for 3D imaging of the intestinal tract of live zebrafish.^91^ Bozycki et al. successfully identified pathological calcium deposits with enhanced brightness in whole-body mouse skeleton using alizarin red S.^92^ Other than designing fluorophores with better optical properties, there is also an increase in demand for dyes that emit fluorescence in the near-infrared region. The longer wavelength emission of the dyes enables better tissue penetration and reduced scattering, which is beneficial for deep tissue imaging.^93^ An example of a near-infrared fluorescent dye is the Hexamethylsilole Cyanine (HMSiR) dye.^94^ Its spontaneous blinking nature and long wavelength excitation property enable a low phototoxic methodology for live 3D localization-based imaging.^22^ Grimm et al. also recently made progress in the development of rhodamine-related dyes, which provided insights for future fluorescence probe designs.^95^ Owing to their small size, fluorescence dyes were also utilized for intracellular imaging and observing dynamic intracellular activities.^96^ However, when facing certain types of protein labeling, the specificity was generally low. Hence, fluorescence dyes were also integrated with genetically encoded probes to expand their range of applications, which will be discussed in the next section.

#### 2.3.2 Genetically Encoded Probes

The discovery of fluorescent protein (FP) in 1962 by Shimomura et al. provided a larger pool of fluorescent tools to be applied in LSFM.^97^ Through gene fusion, specific labeling of a target protein and fluorophore can be achieved with ease.^98^ From the most commonly used green fluorescent protein (GFP) and its variants (EGFP) to spectrally distinct FP that emit orange (mKO), red (mCherry), and far red (mKate2) light,^99^ FP has become a common practice to study the complex cell behaviors and live cell imaging using LSFM.^100,101^ Although the application of FP in LSFM seems highly compatible, the need to perform optical clearing in LSFM often interferes with FP performance. Kirschnick et al. have developed a methodology to evaluate tissue-clearing protocols using a 3D-polymerization cell dispersion technique to determine fluorescence retention.^102^ With this addition to the LSFM toolbox, it is now possible to overcome this limitation.

FP, as compared to fluorescent dyes, usually possesses relatively lower brightness and weak photostability and is often larger in size. Even though FP has been widely used in many LSFM-related research studies, there is a need to improve its performance. As mentioned earlier in the previous section, FP can be integrated with fluorescence dyes to enhance performance.^103^ The development of enzyme-based self-labeling tags (SNAP-Tag,^104^ Halo-Tag^©^^105^), which combines the genetic specificity of FP and the freedom to select fluorescent dyes with better optical properties, has further enabled the advancement in LSFM-related research. By using tetramethylrhodamine (fluorescent dye) with HaloTag (FP), Chen et al. applied it in the target searching process and binding kinetics of the pluripotency regulators in the mouse embryonic stem cells^106^ and Liu et al. have demonstrated the capability of single-molecule tracking of transcription factor Sox2.^107^ Mentioned earlier, the HMSiR dye can serve as an excellent fluorescent dye but lacked specific protein binding properties. In the work of Urano et al., they successfully addressed this problem by integrating Halo and SNAP-tag to HMSiR, which enabled imaging of microtubules in living cells.^108^

Most conventional FPs are known to possess low photostability, which greatly limits their applications in fluorescence microscopy. There is still much ongoing research regarding the improvement of genetically encoded probes, many of which demonstrate superior selectivity for specific proteins for different applications. One such example is the application of OxLight1 that enables accurate reports of the dynamics of endogenous orexins in the mouse brain.^109^ In recent years, several improved genetically encoded probes have found applications in super-resolution microscopy. Hirano et al. presented StayGold, a variant of GFP, which possesses enhanced photostability as compared to existing FPs.^110^ Ning et al. also developed a new bright red FP that is less toxic than conventional red FP such as mCherry or mKate2 for neuron visualization.^111^ Laviv et al. designed a red fluorescence protein with a large Stokes shift that can be simultaneously used with EGFP-based sensors for dual imaging of signaling molecules in single dendritic spines.^112^ Although the use of these novel genetically encoded probes has yet to find applications in LSFM, we foresee that more genetically encoded probes that enable improvement in current LSFM methodology will be available in the future.

#### 2.3.3 Nanoparticle-Based Probes

The discovery of quantum dots (QDs) in the 1980s provided another alternative to consider when choosing appropriate fluorescent probes for varying applications. QDs are nanocrystals typically in the size range of 2–50 nm that, when excited, produce fluorescence at a wavelength based on the size of the particle.^113^ Due to their excellent photostability and enhanced brightness, QDs were ideal fluorescent labels for long-term single-molecule tracking candidates.^114,115^ QDs were also investigated for their potential application as a labeling agent in zebrafish embryos.^116^ However, the downside of QDs is that they show poor cell permeability, which poses a problem during intracellular imaging.^117^ There are also reports regarding cell toxicity in response to the breakdown of the particle.^118^ Nevertheless, with some modification of QD surface morphology, Dennis et al. were able to facilitate the endosomal uptake of the QD entity for intracellular imaging.^119^ The use of QDs in LSFM-related applications was validated by works including single particle tracking^120,121^ and noninvasive in vivo imaging of mouse tumors.^122^ Another interesting branch of nanoparticle-based probes is carbon dot (CD). Fluorescent CD, unlike QD, does not contain any metal in its composition and possess good biocompatibility, low toxicity, and most importantly, they can easily enter cells and interact with specific compartments/biochemical due to their small size.^123^ They can be easily functionalized for specific targeting of intracellular components and intracellular chemicals.^124^ However, there has been no reported work regarding the application of CD in LSFM. We remain hopeful that CD could surpass QD and find its place in LSFM in the future.

#### 2.3.4 Strategies for Possible Future Applications in LSFM

We have discussed several commonly used fluorescence probes that were used in LSFM or potential candidates that may find applications in future development of LSFM. One may notice that fluorescence probes used in different applications were often catered for type-specific labeling; for example, Lesiak et al. use HMSiR680-ME dye that is lysosome specific for visualizing the interaction between lysosome and mitochondria,^125^ while Doll et al. perform real-time tracking in living cells with siRNA labeled with ATTO dyes.^126^ In order to select the most suitable probe for type-specific applications, one may have first to determine the desired target, such as proteins,^127^ lipids,^128^ or carbohydrates.^129^ After locking on to a specific target, the next question to answer is the kind of bridging strategies we should use for signal optimization, such as self-labeling tags^104,105^ or via chemical reactions such as biorthogonal conjugations.^130^ With the necessary parameters determined, there will be a better chance of satisfactory results.

As the development for LSFM continues to expand, many techniques are being developed that add to its arsenal. Chen et al. presented the idea of expansion microscopy (ExM) in 2015, which greatly improves the capabilities of LSFM.^131^ Briefly, ExM involves physically expanding the samples using polyelectrolyte hydrogel via swelling and stopping it via chemical treatments.^132^ In order to apply fluorescence labeling to ExM, it is important to ensure that the probes can accommodate extensive chemical treatments. Wen et al. use a series of stabilizer-containing multifunctional molecules that can withstand vigorous chemical treatment during sample preparation and retain fluorophore photostability.^133^ Even though many of the mentioned strategies have yet to find applications in LSFM, we remain positive that in the near future, as LSFM development continues to progress, these strategies may soon find a place to support the advancement of LSFM.

## 3 Applications of Light Sheet Fluorescence Microscopy

In the previous section, we discussed the general improvements of LSFM in a specific way. We now move our focus back to LSFM for biological applications. It should be known that in order to pry deeper into subcellular level observations, it is unavoidable to concentrate on the system setup with a high NA detection objective lens because of the enhanced axial resolution. In this section, we discuss the leading groups that combine several techniques to cater to specific applications (mostly in subcellular imaging) with a focus on system setups that are equipped with high NA detection objectives. We hope to provide an overview of modern LSFM techniques for our readers to have a clearer picture of the upgrades or strategies required for varying biological applications. We also have to stress that there is no “best” setup of LSFM, as different applications require varying techniques to yield the desired results. In the following sections, NA values of the detection objective used in several examples will be shown.

### 3.1 Live Imaging in 5D

**Figure 4:**
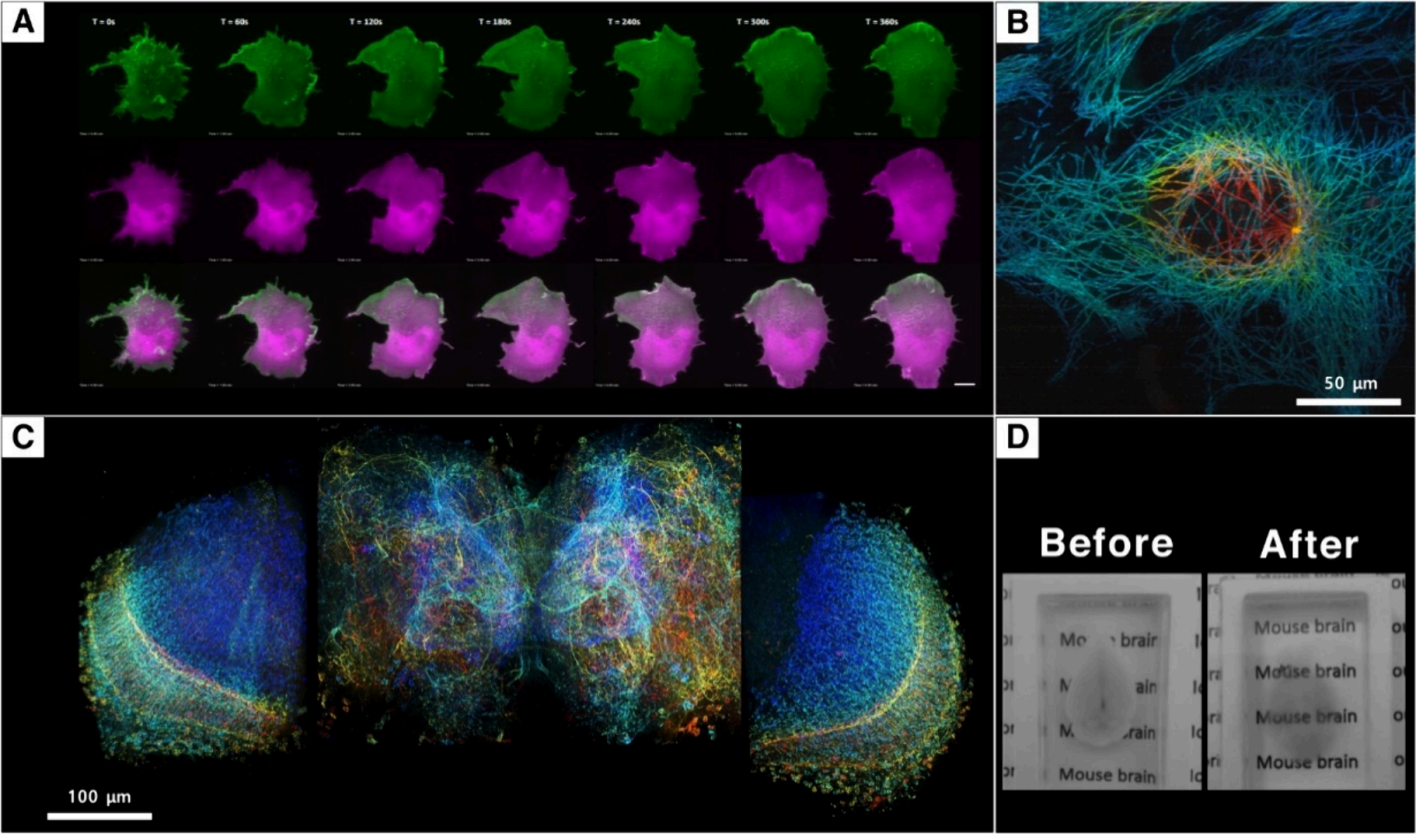
Application of LSFM. (A) Application of LSFM in live imaging in 5D. The figure shows the time lapse images of the extension membrane, which had a processing mobility between protrusion and retraction on the filopodial area. During membrane ruffling processing, once the trigger is on (rapamycin added); the signals originally resided at the nucleus will go to the cell membrane and ruffling will happen. The green color shows LFC-EYFP-CAAX membrane signals; the magenta color shows RFiSH distribution through the cell. The bottom row shows the merged ones. Refer to Supporting Information S1 and S2 for the movie. (B) Application of LSFM in live imaging in 5D. The image of a microtubule during localization. Refer to Supporting Information S3 for movie. (C) Application of LSFM in large sample analysis. High-resolution image of whole Drosophila brain. Different colors depict various regions. (D) Application of LSFM in sample manipulation (mouse brain). Before and after images of mouse brain undergoing sample clearing.

The significance of live imaging in LSFM has always been paramount in biological research since the ability to visualize dynamic processes on the subcellular level within live samples in real-time can further elevate our understanding of developmental mechanisms and cellular interactions. Many researchers have already displayed such capability, but most of them are restricted within the fourth dimension (tracking changes over time).^134,135^ The addition of the fifth dimension, which involves simultaneously observing multiple fluorophores or molecular markers, has a significantly elevated LSFM capability. By tracking live samples in five dimensions (5D), it enables the visualization of dynamic events within living organisms with spatial, temporal, and spectral precision.^136^Figure 4A,B shows some examples of 5D live imaging; for the full movie of Figure 4A please refer to Supporting Information S1 and Supporting Information S2. In this two-color experiment, the camera area of 160 × 832 pixels at a pixel resolution of 102 × 102 nm was used for imaging. The exposure time for each frame is 19 ms, the sample scanning step size is 0.27 μm, and 131 planes of images are used for each of the two channels to conduct volumetric imaging with a 5s acquisition time and 1s pause time^137^ (NA 1.1). For the full movie of Figure 4B please refer to Supporting Information S3. However, challenges persist in managing the volume of data generated as well as achieving rapid acquisition speeds required for capturing fast biological processes. Not to mention, it is also a challenging task to maintain an optimal resolution and contrast across multiple dimensions. Nevertheless, different methods that are equipped with advanced optics, imaging protocols, and computational algorithms have managed to address these challenges and fully harness the potential of 5D live imaging in LSFM. Even though the protocols for live imaging in 5D for studying dynamic biological processes in living organisms have not been ideally optimized, most of the current methodologies are able to capture spatial, temporal, and spectral information simultaneously, which offers unparalleled insights into cellular processes and developmental changes.^138^ Future advancements might focus on improving imaging speed, reducing phototoxicity, and enhancing computational tools for data analysis. The evolution of five-dimensional live imaging in LSFM is poised to unlock new frontiers in understanding complex biological systems.

### 3.2 Super-Resolution Imaging

The pursuit of super-resolution imaging has always been one of the ultimate goals in LSFM. It enables researchers to surpass the Abbe diffraction limit and achieve higher spatial resolution in biological samples. The visualization of finer details within cellular structures and subcellular components could provide a more comprehensive understanding of biological systems. With the improvement in resolution for live 3D imaging,^22^ the study of nanoscale structures and intricate biological interactions such as cellular growth and tissue dynamics during growth of crops^139^ (NA 1.0/1.1) or mapping out the architecture of neural circuits^60^ (NA 1.1) are now possible. However, there remain several issues in super-resolution LSFM that researchers are actively trying to resolve. The increased complexity in imaging setups, longer acquisition times, and potential phototoxicity due to prolonged exposure to high-intensity illumination are some notable problems. Overcoming these challenges requires advancements in specialized optics and innovative imaging strategies without compromising sample viability. Tsai et al. introduced a tiling lattice light sheet method that involves applying different binary phase maps to the binary SLM used in a lattice light sheet microscope. This modification enhanced the overall resolution with doubled imaging speed^140^ (NA 1.1). In another example, Gustavsson et al. managed to capture a super-resolution image of mammalian cell mitochondria in tens of nanometer localization precision with the novel implementation of tilted light sheet illumination with long axial range PSF^141^ (NA 1.4). To deal with the problem of phtotoxicity, Gao et al. suggested a strategy that involves the use of Bessel beam SPIM, which greatly reduced phototoxicity as compared to other methods when imaging rapid morphological changes in *D. discoideum* cells with super resolution^142^ (NA 1.1). Another way to enhance resolution is the integration of expansion microscopy and lattice LSFM. Gao et al. proposed a method for the imaging of neurons in the mouse cortex or the whole *Drosophila* brain. They successfully capture the images of neurons down to their molecular constituents, such as synaptic proteins, over large volumes^143^ (NA 1.1). The journey to achieve super-resolution in LSFM is still in its early stage, despite that many existing methods have already achieved this goal. Future studies should concentrate on refining these methods within LSFM setups, aiming to achieve better penetration depths, reduced acquisition times, and improved compatibility with live imaging. Another direction for improvement is to mitigate phototoxicity, increase imaging speeds without sacrificing resolution, and enable real-time observations of dynamic biological processes within large intact samples. We hope that the journey to achieve super-resolution will continue to push the boundaries of what is feasible and empower deeper insights into complex biological phenomena.

### 3.3 Large Sample Analysis

Large sample analysis in LSFM holds immense significance due to its ability to provide a comprehensive view of complex biological systems such as zebrafish.^144^ Analysis of large samples offers a more accurate and complete representation of biological structures and processes within their natural context, which enables a deeper understanding of spatial relationships, cellular interactions, and dynamic behaviors across tissues and organisms.^140^Figure 4C is an image of the whole *Drosophila* brain with different colors that were used to represent different regions. To proceed with further discussions in this section, all of the large samples we mentioned are samples with sizes larger than 100 μm. To study large samples is advantageous because the preservation of structural integrity can be achieved. The study of intact tissues and organs^6^ and observing dynamic processes in these samples over extended periods^145^ are also a few of the reasons why scientists are pursuing large sample analyses using LSFM. In order to visualize large samples with LSFM, there are several challenges that remain to be addressed. One has to carefully balance the trade-off between the resolution, sample size, and instrumental constraints. In the past, the resolution and image quality are often sacrificed to accommodate the analysis of large samples. Many solutions were provided during recent development that mostly focused on sample handling or instrumental adjustments. Lu et al. introduced tiling lattice light sheet microscopy that makes use of optical tiling instead of mechanical tiling. This method greatly improves resolution and image quality when analyzing large or expanded samples^146^ (NA 1.1). The innovative integration of macro photography with LSFM introduced by Lee et al. enhanced the effective resolution of a 3.7 cm thick whole brain to 300 nm by 4 times tissue expansion, even though with low NA detection objective^9^ (NA 0.3). Instrumental adjustments such as optical tilting^140,146^ and removal of illumination objective lens^147^ further improve the versatility of the LSFM system for large sample analysis. One can even design and fabricate customized sample mounting platforms that are optimized for specific applications involving large samples^148^ (NA 0.3). Analyzing large samples is very promising in uncovering the intricate workings of biological systems in their most natural form. However, with the increase in the size of the sample, the NA value of the detection objective has to be lowered significantly to avoid collision of the lens with the sample. Despite the challenges, advancements in computational tools, optics, and imaging strategies continue to expand LSFM’s capabilities. Future advancements may focus on enhancing imaging depths, improving resolution across larger volumes, and developing more efficient data processing methods.^149^ Another potential advancement in LSFM is the integration of AO, mentioned earlier in this Review. Briefly, AO can be used to enhance image resolution by correcting sample-induced distortion.^23^ As LSFM evolves, we believe that researchers can come up with a novel methodology for the analysis of intricate biological processes that enable discoveries in developmental biology and disease mechanisms and provide insights into the functioning of complex biological systems.

### 3.4 Sample Manipulation

From previous sections, we know that sample manipulation techniques for LSFM, particularly sample clearing methods, are essential because they can maximize imaging depth and resolution within biological samples. A sample clearing process involves treating biological samples with clearing agents or protocol to minimize the detrimental effects of light scattering while enabling light to penetrate deeper into large and intact samples for clearer visualization (Figure 4D). Common clearing methods are classified into two major groups: organic solvents-based (BBAB,^150^ 3DISCO,^151^ uDISCO^152^) or water-based, while water-based methods are further divided into hydrogel embedding-based (CLARITY^153^), immersion-based (SeeDB,^154^ SeeDB2^155^), and hyperhydration-based (CUBIC1,^156^ CUBIC2^157^). Comparative studies were also conducted to evaluate various clearing methods and their organ-specific applications.^158^ Other studies that involve optimization and integration of sample clearing methods to LSFM were also developed.^159,160^ In particular, Chen et al. developed a versatile tiling light sheet microscope for all tissue-clearing methods and successfully produced 3D images with subcellular resolution.^34^ In addition to clearing methods, ExM was popular when it was used for sample manipulation. ExM, which physically expands the sample isotropically, enhances image resolution beyond the limitations of traditional microscopy.^161^ Du̅ring et al. have shown that by integrating ExM with LSFM, they could produce images of large volumes of brain tissues at subcellular resolution.^37^ A similar integrated setup has also been shown to be capable of producing volumetric imaging of virus-infected cells.^162^ We have shown that with the appropriate tools, the capabilities of LSFM could be further improved to enhance image quality. Both of the above-mentioned techniques offer insights into cellular architecture and interactions, which could further assist researchers to have a deeper understanding of biological phenomena. Future development should focus on the simplification of current protocols and further optimize compatibility with LSFM for a better visualization of different biological specimens.

### 3.5 Integration to Other Platforms

**Figure 5:**
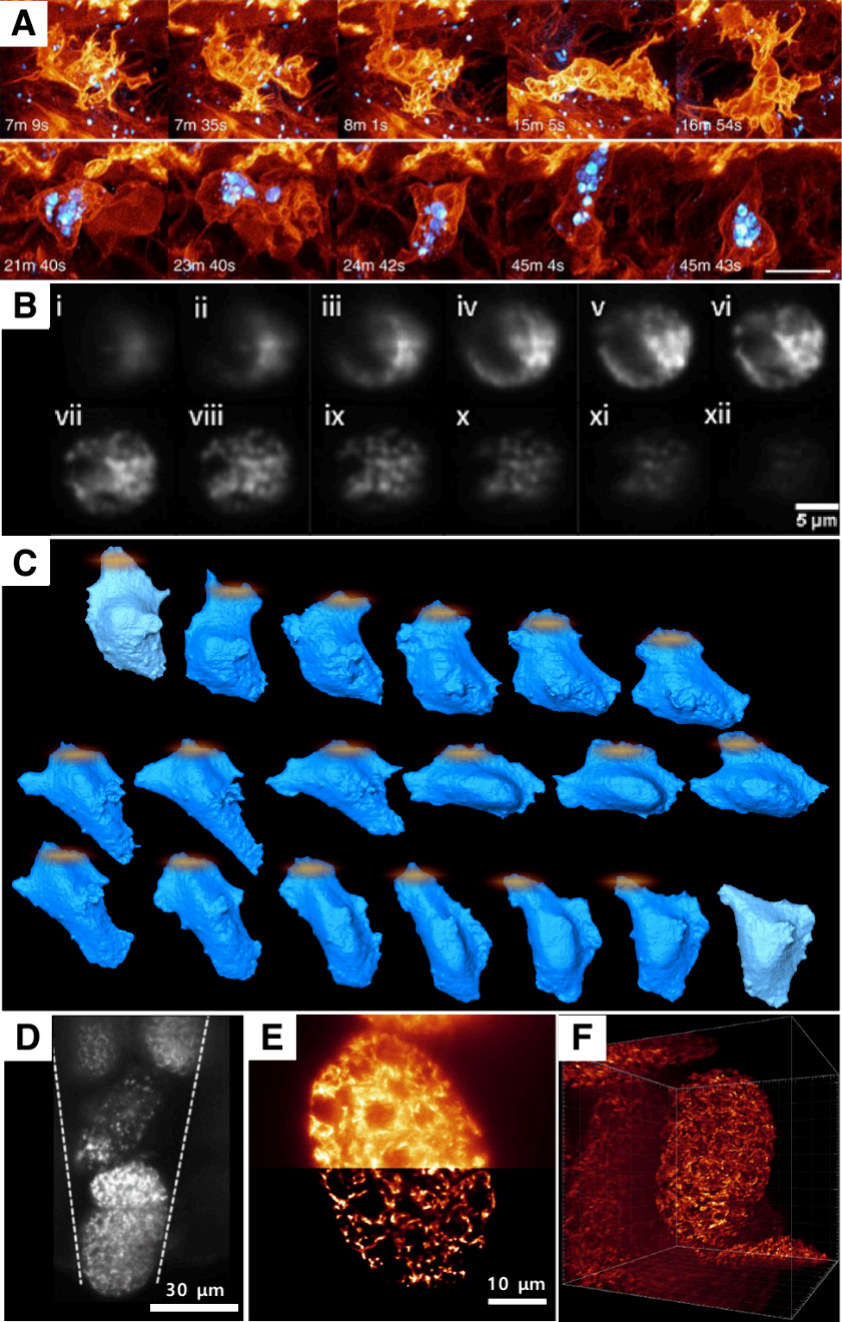
Integration of LSFM to other platforms. (A) Integration of LSFM to adaptive optics. The images show 3D cell migration in vivo, changing morphologies of two different immune cells (top and bottom rows), one showing internalized dextran particles (blue). Scale bar 5 um. Reproduced with permission from ref (^23^). Copyright 2018 American Association for the Advancement of Science. **(B(i)-(xii))** Integration of LSFM to microfluidics. The sequential images of Chinese hamster ovary (CHO) cells flowing through the lightsheet via a microfluidic channel at intervals of time of Δ*t* = 120 ms at a speed 1.49 um/s. Reproduced with permission from ref (^168^). Copyright 2021 Royal Society of Chemistry. (C) Integration of LSFM to optogenetic activation. The series of images show the change in the morphology of cell during of cell migration when excited by a light signal in real time. **(D–F)** Integration of LSFM to microneedle. Illumination of the trapped embryos of *Caenorhabditis elegans*. (D) Image of an embryo trapped by the microneedle. (E) Sliced image *z* = 45 of the first acquired group of images with deconvolution. (F) Reconstructed 3D image of deconvoluted image at time lapse of 10 min. **(D–F)** Reproduced with permission from ref (^170^). Copyright 2022 Royal Society of Chemistry.

We have seen the capabilities of LSFM as a standalone instrument and its extensive range of biological applications in previous sections. Despite LSFM’s prowess as an independent tool, there are many different systems that can be coupled to further amplify its power and versatility. For example, the introduction of AO has solved the issues regarding optical aberration, a common phenomenon in systems with high NA objectives.^16^ Liu et al. took advantage of AO and successfully obtained high-resolution live-cell imaging of subcellular processes such as organelle remodeling during mitosis or cell migration *in vivo* (Figure 5A)^23^ (NA 1.1). Hung et al. developed a new methodology coined AO-SOLEIL (adaptive optics-single-objective lens inclined light sheet microscope) that enables super-resolution imaging of neurons in adult *Drosophila* brains^163^ (NA 1.35). With the joint efforts of researchers, there are now detailed protocols available to integrate AO to LSFM.^164^ Another example is the integration of microfluidic systems to LSFM. The combination of a custom microfluidic system with LSFM brings about numerous advantages in biological imaging. To name a few, one can tailor the microenvironment which enables precise control of the sample environment. Moreover, one can also reduce sample handling complexities, minimize disruptions to delicate specimens, and facilitate long-term observations (Figure 5B).^165−168^ By utilizing these advantages, Meddens et al. obtained high-quality 3D whole-cell images with enhanced localization and significant reduction in photobleaching^24^ (NA 1.2). As mentioned before, in most LSFM systems involving biological applications, the use of high NA objectives is essential; however, through integration to various systems, one can circumvent this exclusive reliance. This broadens the scope of samples and offers more versatility with application-specific setups. By incorporating a multimodal imaging system, simultaneous high-resolution images of the same plane can be obtained in murine embryos, which can facilitate real-time visualization.^18^ Cell manipulation can also be done in real-time when combining LSFM with optogenetic activation (Figure 5C)^169^ (NA 1.1). Other innovative integrations include microneedle for continuous embryonic imaging (Figure 5D-F)^170^ (NA 1.1) and 3D culture visualization^171^ (NA 1.1). In conclusion, the introduction of various systems into LSFM widens the applicability across a variety of biological samples and experimental conditions. Future directions may involve enhancing compatibility with emerging technologies, such as AI for real-time image analysis or novel sample preparation methods. We remain hopeful that these directions aim to optimize LSFM’s adaptability and foster its utilization across a spectrum of biological research, from subcellular investigations to macroscopic imaging and propelling innovative discoveries in diverse scientific fields.

## 4 Conclusion

The future of LSFM development holds promising avenues poised for advancement in various domains. Integration with AO ensures improved image quality by compensating sample-induced aberrations, augmenting LSFM’s capabilities across diverse biological specimens. Moreover, leveraging AI stands as a potential catalyst, aiding in real-time data analysis and enhancing imaging efficiency. While LSFM can benefit from these innovations, the importance of high NA objectives remains crucial, enabling detailed imaging of subcellular structures and providing finer resolution for intricate biological processes. Future developments in LSFM will likely converge on synergizing these advancements, forging a path toward more comprehensive imaging solutions that cater to the intricate demands of biological research.

## Supporting Information Available

**Supporting Information S1**: Movie of membrane ruffling in the color green (MP4)
**Supporting Information S2**: Movie of membrane ruffling in the color magenta (MP4)
**Supporting Information S3**: Movie of microtubule localization (MPG)

## Supplementary Material

jp4c01721_si_002.mp4

jp4c01721_si_003.mpg

## Abbreviation

LSFM: Light Sheet Fluorescence Microscopy
3D: Three Dimensional
SPIM: Single-Plane Illumination Microscope
MuVi-SPIM: Multiview Light Sheet Microscopy
AO: Adaptive Optics
AI: Artificial Intelligence
NA: Numerical Aperture
SLM: Spatial Light Modulator
CMOS: Complementary Metal Oxide Semiconductor
FEP: Fluorinated Ethylene Propylene
2D: Two Dimensional
PSF: Point Spread Function
DAPI: 4′,6-Diamidino-2-Phenylindole
H_2_S: Hydrogen Disulfide
FP: Fluorescent Protein
GFP: Green Fluorescent Protein
EGFP: Enhanced Green Fluorescent Protein
QD: Quantum Dot
CD: Carbon Dot
5D: Five Dimensional
ExM: Expansion Microscopy
AO-SOLEIL: Adaptive Optics-Single-Objective Lens Inclined Light Sheet Microscope
